# Efficacy of OLIF combined with pedicle screw internal fixation for lumbar spinal stenosis on spinal canal changes before and after surgery

**DOI:** 10.1186/s13018-023-04209-2

**Published:** 2023-09-25

**Authors:** Wangbing Xu, Weibing Liu, Faming Zhong, Yu Peng, Xin Liu, Liangkun Yu

**Affiliations:** 1 Affiliated Hospital of Jiangxi University of Chinese Medicine, Nanchang, Jiangxi 330006 China; 2 Jiangxi University of Chinese Medicine, Nanchang, Jiangxi 330004 China; 3 The Fourth Clinical Medical College, Guangzhou University of Chinese Medicine, Shenzhen, Guangdong 518033 China

**Keywords:** Lumbar spinal stenosis, Oblique lumbar interbody fusion, Disc height, Spinal canal cross-sectional area

## Abstract

**Purpose:**

The purpose of the study was to evaluate the efficacy of OLIF combined with pedicle screw internal fixation in the treatment of lumbar spinal stenosis by assessing the changes in spinal canal before and after surgery.

**Methods:**

In this retrospective study, we included sixteen patients who underwent a combination of single-segment OLIF and pedicle screw internal fixation for the treatment of lumbar spinal stenosis at the Affiliated Hospital of Jiangxi University of Chinese Medicine between February 2018 and August 2022. The patients' pre- and post-operative data were compared. Intraoperative bleeding, duration of surgery, visual analogue score (VAS), Oswestry Disability Index (ODI), disc height (DH), cross-sectional area of vertebral canal (CSAVC), cross-sectional area of dural sac (CSADS), cross-sectional area of intervertebral foramen (CSAIF), spinal canal volume (SCV), spinal canal volume expansion rate, lumbar lordosis, and sagittal vertical axis were observed and recorded. The efficacy of OLIF combined with pedicle screw internal fixation for lumbar spinal stenosis on spinal canal changes before and after surgery was summarized.

**Results:**

The results showed that OLIF combined with pedicle screw internal fixation effectively restored disc height and increased the cross-sectional area of the spinal canal. It also had an indirect decompression effect. The intraoperative bleeding and duration of surgery were within acceptable ranges. The VAS and ODI scores significantly improved after surgery, indicating a reduction in pain and improvement in functional disability. The CSAVC, CSADS, CSAIF, SCV, and spinal canal volume expansion rate were all increased postoperatively. Additionally, there was improvement in lumbar lordosis and sagittal vertical axis. We conducted a follow-up of all patients at 1 year after the surgery. The results revealed that the parameter values at 1 year post-surgery showed varying degrees of decrease or increase compared to the immediate postoperative values. However, these values remained statistically significant when compared to the preoperative parameter values (*P *< 0.05).

**Conclusions:**

OLIF combined with pedicle screw internal fixation effectively restores disc height and increases the cross-sectional area of the vertebral canal in patients with LSS, reflecting the indirect decompression effect. Measuring parameters such as DH, CSAVC, CSADS, CSAIF, SCV, and SCV expansion rate before and after surgery provides valuable information for evaluating the efficacy and functional recovery of the lumbar spine in LSS patients treated with OLIF surgery.

## Introduction

Lumbar spinal stenosis (LSS) is a condition characterized by degenerative changes, including facet joint disorder, hypertrophy or calcification of the ligamentum flavum, and disc bulge. These changes lead to compression of the spinal dura mater and nerve roots due to stenosis of the spinal canal or intervertebral foramen [1], typical clinical manifestations of LSS include absence of pain while sitting, symptom improvement with flexion, extensive gait (difficulties with walking), and intermittent claudication. These findings are crucial for clinical diagnosis [2]. According to Kalichman et al. [3], the incidence of acquired lumbar spinal stenosis (LSS) increases with age, with rates rising from 16.0% and 4.0% to 38.8% and 14.3% between the ages of 40 and 60, respectively. In clinical practice, traditional posterior lumbar fusion (PLIF) is considered the "gold standard" for treating LSS. However, PLIF often leads to residual symptoms due to its impact on paravertebral muscles, the dural sac, and nerve roots. Additionally, its ability to correct sagittal balance and lumbar lordosis is not as effective as OLIF [4, 5]. OLIF is a natural space approach between the anterior oblique lateral psoas major muscle and the sheath of great abdominal vessels, which can avoid the complications of OLIF, and has the advantages of less trauma, less blood loss during operation, and short duration of operation [6]. At present, most clinical studies comparing treatments for LSS focus on comparing OLIF and MI-TILF [7–9]. However, these studies mainly focus on clinical parameters such as intraoperative blood loss, operative time, and hospitalization days. Even if they do analyse radiological parameters, it is only a superficial analysis of sagittal sequence parameters such as disc height and lumbar lordotic angle. There is a lack of research on the changes in the spinal canal before and after OLIF combined with pedicle screw fixation, which can reflect the indirect decompression effect of LSS. Therefore, the purpose of this study is to compare the changes in disc height (DH), cross-sectional area of the vertebral canal (CSAVC), cross-sectional area of the spinal canal (CSADC), cross-sectional area of the intervertebral foramen (CSAIF), spinal canal volume (SCV), and SCV expansion rate before and after OLIF combined with pedicle screw fixation in LSS patients. This study aims to reflect the indirect decompression effect of OLIF and highlight its advantages in terms of reduced trauma and shorter operation time.

## Materials and methods

A retrospective analysis was conducted on sixteen patients with mild to moderate lumbar spinal stenosis who underwent OLIF combined with pedicle screw internal fixation at the Affiliated Hospital of Jiangxi University of Traditional Chinese Medicine from February 2018 to August 2022. The patients were selected based on the inclusion and exclusion criteria. Among the participants, there were three males with an average age of 63 years and thirteen females with an average age of 66 years. There were no significant differences between the two groups (*P *> 0.05). Preoperative, postoperative, and last follow-up imaging data were collected for all LSS patients, including anterior and lateral X-ray CT scans and magnetic resonance imaging (MRI) of the lumbar spine.

### Inclusion criteria

(1) The diagnostic criteria for lumbar spinal stenosis (LSS) in the subjects were based on the second edition of the Guidelines for the Diagnosis and Treatment of Degenerative Lumbar Spinal Stenosis (NASS): According to these criteria, all patients exhibited symptoms of neurogenic claudication and experienced pain in the buttocks or lower extremities. These symptoms were aggravated by provocative stimulation of the neurogenic source during upright walking or in certain positions and relieved by palliative measures targeting the neurogenic source, such as forward bending, sitting, or lying down [10]; (2) the classification of lumbar spinal stenosis (LSS) in all patients included in this study aligns with the indications for OLIF surgery, ranging from mild to moderate–severe spinal stenosis. Mild to moderate spinal stenosis is typically defined when the cross-sectional area of the dura mater measures between 75 and 100 mm, while severe spinal stenosis is characterized by a cross-sectional area of less than 75 mm [11, 12]; (3) we confirmed a reduction in intervertebral disc height in all patients, comparing the preoperative disc height of the most severe and mildest symptomatic patients with the disc height of adjacent segments; (4) the anterior and lateral DR radiography and MRI data of the lumbar vertebrae were evaluated before and after the operation, and the results were found to be satisfactory; (5) preoperative X-ray films and lumbar MRI of all subjects confirmed that OLIF could be performed within the natural space between the anterior oblique lateral psoas major muscle and the abdominal great vessel sheath; and (6) the follow-up period for all subjects included in the study was more than 1 year.

### Exclusion criteria

(1) Lumbar spinal stenosis can be caused by various factors such as lumbar trauma, infection, tuberculosis, tumours, and other reasons; (2) it is also observed in patients with bony spinal stenosis, congenital spinal stenosis, and those with space-occupying lesions in the spinal canal [11]; (3) measure the existence of deformity, congenital diseases, hereditary diseases, etc., in the lumbar spine of the surgical segment; and (4) individuals who are afflicted with severe primary diseases and are frail, rendering them unable to withstand surgical intervention.

### Surgical method

#### Preoperative preparation

(1) Conduct a thorough diagnostic assessment to accurately identify and rule out lumbar spinal stenosis resulting from infection, tumour, tuberculosis, and other potential causes; (2) enhance the quality of preoperative X-ray and MRI scans to ensure that the patient's anterior oblique lateral psoas major muscle and the natural gap between the abdominal large vascular sheaths can accommodate OLIF surgery; (3) perform a comprehensive preoperative evaluation to verify that the patient's overall health condition meets the necessary criteria for undergoing general anaesthesia and tracheal intubation during surgery; and (4) enhance the performance of other relevant tests to provide a comprehensive assessment of the patient's medical status.

#### Surgical procedure

All patients underwent general anaesthesia with endotracheal intubation. Following successful anaesthesia, patients were positioned correctly in the lateral position, with the lumbar bridge adjusted to an appropriate height. The knee and hip joints were flexed, and folding cushions were utilized to isolate and protect the lower limbs. Subsequently, routine disinfection and placement of sterile towels were performed. Using C-arm fluoroscopy, the patient's body position is adjusted until obtaining a standard X-ray image of the target intervertebral space and fixing the patient's position. An oblique incision, approximately 4–6 cm in length, is made in front of the midpoint of the target space projection. This incision exposes the subcutaneous soft tissue and allows for the sequential blunt separation of the corresponding muscle tissue layers. The dissection continues obliquely from the front towards the peritoneum, penetrating the intervertebral space through the natural gap between the major psoas muscle and the abdominal vascular sheath. During the process of intervertebral disc exposure, by utilizing the magnification effect of the endoscope, we can clearly identify the position of the intervertebral disc. Carefully, we insert a tube into the tubular channel system and connect the light source to ensure optimal visibility of the target intervertebral disc within the channel. Additionally, we use a vertebral distractor, starting with larger sizes and gradually reducing, to meticulously clean the intervertebral disc tissue using long-handled nucleus rongeurs. Then, the residual intervertebral disc tissue on the endplate is carefully removed using a double-sided scraper. During the procedure, it is observed that there is no tension on the nerve root, indicating that the compressed dural sac is fully expanded, and the lateral saphenous fossa is completely opened. We begin the process of bone grafting and intervertebral fusion on the surgical segment, starting with a trial model from small to large sizes. After the trial is completed, we once again utilize the magnification effect of the endoscope to accurately place an appropriately sized allograft bone cage into the target intervertebral space and fill it. Under C-arm fluoroscopy, the height of the intervertebral space is restored in the anterior and lateral positions, with the cage positioned at the centre of its location.

Following the anterior procedure, the patient was positioned prone. Two screws were then implanted into the pedicles of the patient's upper and lower vertebral bodies at the operative segment, ensuring that the height of the intervertebral space was not compressed. Titanium rods were used to stabilize and fix the screws. After thorough flushing, the surgical instruments and gauze were meticulously counted to ensure accuracy. Once bleeding was controlled, a drainage tube was placed, and the incision was closed layer by layer using sutures.

During this surgical procedure, the magnification effect of the endoscope played a crucial role, particularly in the important steps of intervertebral disc removal, bone grafting, and intervertebral fusion. The endoscopic technology provided us with high-definition images, allowing us to observe the surgical area clearly. It also helped us accurately identify vital structures such as nerve roots and the spinal cord, enabling us to better protect them from injury. Additionally, the endoscope provided enhanced visibility, allowing us to detect hidden issues and take prompt measures.

#### Postoperative management

Postoperative care was provided according to standard nursing protocols, including administration of anti-inflammatory, analgesic, stomach protection, and nutritional support medications for symptomatic treatment. Wound healing was closely monitored. On the 3rd day after the surgery, patients underwent routine anterior and lateral reexaminations of the lumbar spine. They were encouraged to start mobilizing and exercising their lumbar spine immediately after getting out of bed. However, excessive weight bearing on the waist was strictly prohibited for the first 3 months after discharge. Patients were advised to wear a waist immobilization belt (JIHENGXIEBEI ID: 20180183) when getting out of bed.

### Clinical evaluation and imaging parameter measurement method

**Fig. 1:**
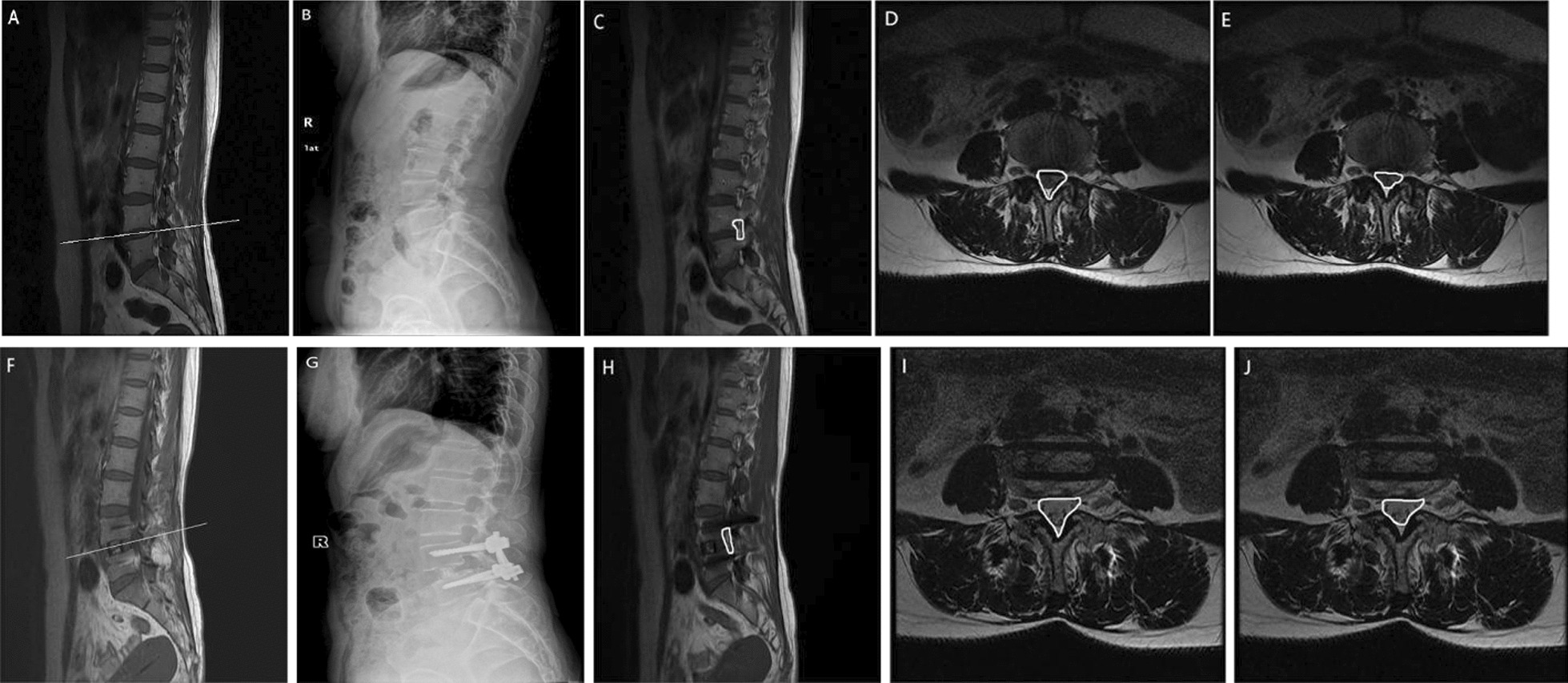
**A** Preoperative surgical segment; **B** preoperative the disc height; **C** preoperative cross-sectional area of intervertebral foramen; **D** preoperative cross-sectional area of the vertebral canal; **E** preoperative cross-sectional area of the dural sac; **F** segment after operation; **G** postoperative the disc height; **H** cross-sectional area of intervertebral foramen after the procedure; **I** cross-sectional area of the vertebral canal after operation; and **J** cross-sectional area of dural sac after the operation

(1) The severity of lumbar pain and dysfunction was evaluated using the visual analogue score (VAS) [13] and the Oswestry Disability Index (ODI) [14] before and after the operation, respectively; (2) the lumbar spine was scanned using the Discovery MR750 3.0T superconducting MRI system, with a fast spin-echo (TSE) scanning sequence. The sagittal and axial positions of the lumbar spine were observed, with the scanning parameters set to T2W1 (TR: 3000 ms and TE: 118.6 ms); (3) DH measurement: The average height of the anterior and posterior edges of the superior vertebral inferior endplate and the inferior vertebral superior endplate on anterior and lateral DR (digital radiography) films of the lumbar vertebrae [6]; (4) CSAVC measurement: On MRI T2WI (T2-weighted imaging), the effective lacuna of the spinal canal area was measured using the posterior edge of the intervertebral disc, the anterior border of the ligamentum flavum, and the inner edge of the pedicle on both sides [6, 15]; (5) CSADS measurement: Dural sac edges were taken as the boundary to delimit the area on T2WI axial images [6, 15]; (6) CSAIF measurement: The intervertebral foramen was wholly displayed in the sagittal position of lumbar MRI [15, 16]; (7) LL measurement: The angle between the upper endplate of L1 vertebrae and the upper endplate of S1 vertebrae was measured on the anterior and lateral DR films of lumbar vertebrae. SVA measurement: The vertical distance from the C7 plumb line to the posterior upper angle of the S1 endplate was measured [17]; (8) SCV measurement: The SCV measurement is based on the concept of cylindrical volume measurement. It is obtained by multiplying the spinal canal area at the midpoint level of the intervertebral space by the height of the intervertebral space [18]; and (9) the expansion rate of SCV is calculated using the formula: (postoperative SCV/preoperative SCV) × 100% [6]. This calculation is used to evaluate the relationship between imaging and clinical results. To ensure accuracy, the values of CSAVC, CSADS, and CSAIF before and after the operation were independently measured three times by two chief physicians using ImageJ-win64 software. The results were then averaged. Typical cases are shown in Fig. 1.

### Statistical analysis

In this study, the data analysis was conducted using SPSS 23.0 statistical software. The surgical blood loss and mean operation time were tested using a single-sample t-test. The DH, CSAVC, CSADS, CSAIF, and SCV parameters were compared using a paired sample *t*-test. To visually represent the measured values of DH and CSAVC before the operation, 1 month after the procedure, and 1 year after the process, line charts with error bars were created. Furthermore, a Pearson correlation analysis was performed to examine the relationship between the decrease in ODI and the increase in DH, as well as the expansion rate of SCV before and after the operation. All parameter results were expressed as "mean ± standard deviation" (*x̅* ± *s*). A *P* value of less than 0.05 was considered statistically significant.

## Results

Table 1 summarizes the basic information of the patients included in the study. The mean age of the patients was 65.4±9.0 years, with a total of 16 participants consisting of three males and 13 females. The average BMI index was 23.3±2.1 Kg/m^2^. In terms of chronic diseases, there were four cases of hypertension, four cases of diabetes, four cases of hypertension and diabetes, and two cases of hypertension and heart disease among the patients. Regarding the type of spinal stenosis, there were four cases classified as mild, 10 cases classified as moderate, and two cases classified as severe. The surgical levels varied among the patients, with 1 case (6.25%) at the L2–L3 level, 4 cases (25%) at the L3–L4 level, and 11 cases (68.75%) at the L4–L5 level. The surgical levels varied among the patients, with 1 case (6.25%) at the L2–L3 level, 4 cases (25%) at the L3–L4 level, and 11 cases (68.75%) at the L4–L5 level. Lumbar instability was observed in 68.75% of the surgical levels, with two cases at the L3–L4 level and nine cases at the L4–L5 level. In terms of complications, 2 cases (12.5%) of cage subsidence and 1 case (6.25%) of endplate injury were observed among the patients. This information provides an overview of the demographic and clinical characteristics of the patients involved in the study.

**Table 1:** Basic patient information materials

	Value
Number of patients, *n*	16
Mean age, year (range)	65.4 ± 9.0
Sex, F/M	13/3
Mean BMI, kg/m^2^	23.3 ± 2.1
*Chronic disease, n (%)*	
Hypertensive	4 (25)
Diabetes	4 (25)
Hypertensive and diabetes	4 (25)
Hypertensive and heart attack	2 (12.5)
*Disease type, n (%)*	
Mild spinal stenosis	4 (25)
Moderate spinal stenosis	10 (62.5)
Severe spinal stenosis	2 (12.5)
*Surgical level, n (%)*	
L2–L3	1 (6.25)
L3–L4	4 (25)
L4–L5	11(68.75)
Lumbar spine instability at suigical level, *n* (%)	11(68.75)
Mean cage height, mm (range)	12.5(11–14)
*Complications, n (%)*	
Cage subsidence	2 (12.5)
Endplate injury	1 (6.25)

Continuous data are shown as mean ± SD

Between 2018 and 2022, we incorporated seven studies on the treatment of lumbar spinal stenosis patients with OLIF indirect decompression surgery (Table 2). The first four of these were retrospective studies, and the last three were comparative studies between the OLIF and MIS-TILF surgeries. Basic information from these studies includes the authors' names, publication years, the number of patients, functional outcomes, radiological outcome parameters, and additional notes (nature of the study). Shimizu et al.'s [6] retrospective cohort study in 2020 involved 42 patients, assessing the improvement of JOA by the OLIF surgery, and evaluated through measuring radiological parameters such as CSAVC and DH. In Gajjar et al.'s [19] retrospective cohort study in 2021, 37 patients were studied, assessing the effect of OLIF surgery on the modified Macnab criteria, and evaluated through measuring radiological parameters such as DH, foraminal height, and CSAVC. Tseng et al.'s [20] retrospective cohort study in 2022 involved 33 patients, assessing the effect of OLIF surgery on the relief of VAS and improvement of ODI, and evaluated through measuring radiological parameters such as DH, foraminal height, and lumbar lordotic angle. Jia et al.'s [21] retrospective cohort study in 2022 studied 10 patients, assessing the effect of OLIF surgery on the relief of VAS and improvement of ODI, and also compared non-radiological parameters such as preoperative and postoperative claudication distance.

**Table 2:** The basic information of seven studies conducted from 2018 to 2022 on the use of OLIF indirect decompression surgery to treat patients with lumbar spinal stenosis

Author	Year	Number of patient	Outcome functional	Radiological outcome parameters	Remark
Shimizu et al. [6]	2020	42	JOA improvement	Disc height (DH) and cross-sectional area of the vertebral canal (CSAVC)	Retrospective cohort study
Gajjar et al. [19]	2021	37	Modified macnab criteria	Disc height (DH), foraminal height, and cross-sectional area of the vertebral canal (CSAVC)	Retrospective cohort study
Tseng et al. [20]	2022	33	VAS relief and ODI improvemen	Disc height (DH), foraminal height, and lumbar lordosis	Retrospective cohort study
Jia et al. [21]	2022	10	VAS relief and ODI improvemen	Non-radiological parameters, but with a comparison of preoperative and postoperative limp distance	Retrospective cohort study
Gao et al. [9]	2022	113	VAS relief and ODI improvemen	Disc height (DH) and foraminal height	Comparative study of OLIF and MIS-TILF
Lin et al. [15]	2018	25	VAS relief and ODI improvemen	Disc height (DH) and the cross-sectional area of intervertebral foramen (CSAIF)	Comparative study of OLIF and MIS-TILF
Zhu et al. [8]	2021	137	VAS relief and ODI improvemen	Disc height (DH) and lumbar lordosis angle (LLA)	Comparative study of OLIF and MIS-TILF

The table presents information from seven previous studies. Each row represents a study and provides details such as authors' names, publication year, number of patients, functional outcomes (e.g. VAS scores or ODI scores), radiographic outcome parameters (e.g. disc height and the cross-sectional area of vertebral canal), and some remarks (e.g. retrospective cohort study and comparative study of OLIF and MIS-TILF)

In terms of comparative studies, Gao et al.'s [9] study in 2022 compared OLIF and MIS-TILF surgeries in 113 patients, assessing the effect of the two surgeries on the relief of VAS and improvement of ODI, and evaluated through measuring radiological parameters such as disc height (DH) and foraminal height. Lin et al.'s [15] study in 2018 compared OLIF and MIS-TILF surgeries in 25 patients, assessing the effect of the two surgeries on the relief of VAS and improvement of ODI, and evaluated through measuring radiological parameters such as disc height (DH) and cross-sectional area of the intervertebral foramen (CSAIF). Zhu et al.'s [8] study in 2021 compared OLIF and MIS-TILF surgeries in 137 patients, assessing the effect of the two surgeries on the relief of VAS and improvement of ODI, and evaluated through measuring radiological parameters such as disc height (DH) and lumbar lordotic angle (LLA). These studies have all affirmed the positive effects of the OLIF procedure in treating patients with lumbar spinal stenosis, whether in terms of functional improvements (such as VAS relief and ODI improvement) or improvements in radiological parameters (such as DH and CSAVC). In addition, comparative studies between the OLIF procedure and the MIS-TILF procedure have also shown similarities in their treatment outcomes.

### Clinical outcomes

1. The mean intraoperative blood loss (43.1 ± 6.3) ml, mean duration of surgery (105.2 ± 7.4) min (the average surgical time calculated in our study refers to the duration from incision to suture, excluding the time spent on pre-anaesthesia, patient positioning during the operation, disinfection, and towel laying.), preoperative VAS of low back pain (6.76 ± 0.59), postoperative VAS (2.15 ± 0.36), preoperative ODI (67.93 ± 3.02), and postoperative ODI (27.40 ± 2.39) for all patients, and the VAS and the ODI were significantly reduced compared to preoperative, with statistically significant differences (*p *< 0.01) (Table 3).
2. Table 4 provides information on the comparison of LL (lumbar lordosis) and SVA (sagittal vertical axis) values before and after the surgical procedure. The LL values in the postoperative group were found to be significantly higher than those in the preoperative group, with statistical significance (*P *< 0.01). This indicates an improvement in lumbar lordosis following the surgery. On the other hand, the SVA values in the postoperative group were substantially lower than those in the preoperative group, with statistical significance (*P *< 0.01). This suggests a reduction in sagittal vertical axis, indicating improved overall spinal alignment. These findings demonstrate the positive impact of the surgical procedure on both lumbar lordosis and sagittal vertical axis, leading to improved spinal alignment and potentially better outcomes for the patients.

**Table 3:** Comparison of changes in preoperative and postoperative visual analogue score (VAS) and dysfunction index (ODI) scores (*n *= 16)

Parameters	Preop (*n *= 16)	Postop (*n *= 16)	*p* value
VAS	6.76 ± 0.59	2.15 ± 0.36	0.00
ODI	67.93 ± 3.02	27.40 ± 2.39	0.00

Postoperative VAS and ODI scores were both significantly lower than preoperative, with statistically significant differences (*p *< 0.01)

**Table 4:** Comparison of changes in preoperative and postoperative sagittal imaging parameters of lumbar lordosis (LL) and sagittal vertical

Parameters	Preope (*n *= 16)	Postope (*n *= 16)	*p* value
LL (°)	34.6 ± 6.7	48.0 ± 5.2	0.000
SVA (mm)	53.6 ± 6.0	24.0 ± 3.3	0.003

LL values were more significantly greater in the postoperative group compared to the preoperative group, with a statistically significant difference (*P *< 0.01), and SVA values were substantially lower in the postoperative group compared to the preoperative group, with a statistically significant difference (*P *< 0.01)

### Evaluation of radiological parameters

**Fig. 2:**
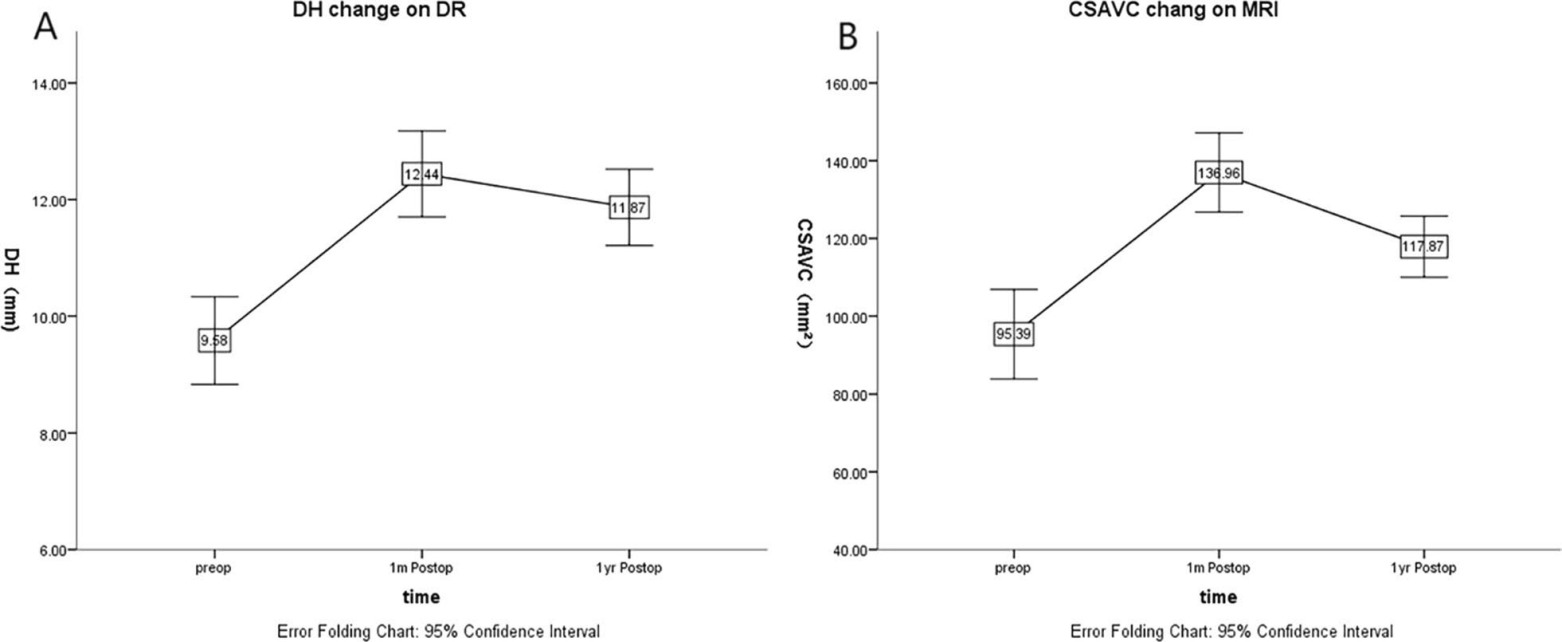
DH (the disc height) change on DR and CSAVC (the cross-sectional area of vertebral canal) change on MRI. **A** A plot of significant change in height on DR preoperative, 1 month postoperative, and 1 year postoperative after DH and **B** a plot of significant change in the area on MRI preoperative, 1 month postoperative, and 1 year postoperative after CSAVC

**Fig. 3:**
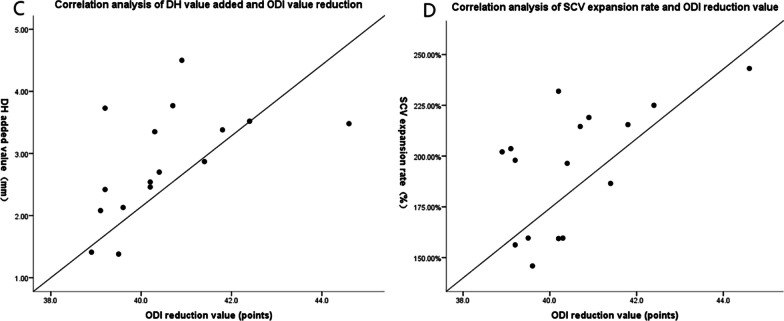
Person correlation analysis of the increase of DH and the expansion of SCV and the decrease of ODI, respectively. **C** The correlation analysis between the increased value of DH and the reduced value of ODI as shown, with a significant correlation (*r *= 0.535, *p *= 0.033) and **D** the correlation analysis between the expansion rate of SCV and the reduced value of ODI as shown, with a significant correlation (*r *= 0.586, *p *= 0.017), *p *< 0.05

1. Table 5 presents a comparison of various parameters before and after the surgical procedure, including disc height (DH), cross-sectional area of the vertebral canal (CSAVC), cross-sectional area of the dural sac (CSADS), cross-sectional area of intervertebral foramen (CSAIF), and spinal canal volume (SCV). The measured parameters after the procedure were found to be increased compared to those before the procedure, and this difference was statistically significant (*P *< 0.05). These findings indicate that the surgical procedure led to an improvement in disc height, increased cross-sectional areas of the vertebral canal, dural sac, and intervertebral foramen, as well as an increase in spinal canal volume. These changes suggest a positive effect on spinal anatomy and potentially improved spinal function. The statistically significant differences observed in these parameters further support the positive impact of the surgical procedure on spinal structure and function (Table 5).
2. Figure 2 illustrates the significant changes in disc height (DH) and cross-sectional area of the vertebral canal (CSAVC) in patients with lumbar spinal stenosis (LSS) before, 1 month, and 1 year after undergoing an oblique lumbar interbody fusion (OLIF) combined with pedicle screw fixation procedure. The preoperative measurements of DH were 9.58 ± 1.40 mm. After 1 month, the measurements increased to 12.44 ± 1.38 mm, indicating a significant improvement in disc height. At 1 year postoperative, the measurements slightly decreased to 11.87 ± 1.22 mm, but still remained higher than the preoperative measurements. Similarly, the preoperative measurements of CSAVC were 95.39 ± 21.51 mm^2^. After 1 month, the measurements significantly increased to 136.96 ± 19.08 mm^2^, indicating an enlargement of the cross-sectional area of the vertebral canal. At 1 year postoperative, the measurements decreased to 117.87 ± 14.69 mm^2^, but were still higher than the preoperative measurements. These line graphs with error bars (representing the 95% confidence interval) visually demonstrate the significant changes in DH and CSAVC following the OLIF procedure with pedicle screw fixation. The upward trend in both parameters indicates an improvement in disc height and an increase in the cross-sectional area of the vertebral canal, supporting the positive outcomes of the surgical intervention for LSS patients (Fig. 2).
3. Figure 3 demonstrates the correlation between functional lumbar recovery, disc height (DH) increase, and spinal canal volume (SCV) expansion in patients with lumbar spinal stenosis (LSS) after undergoing an oblique lumbar interbody fusion (OLIF) procedure. The measurements of DH and SCV were obtained using the ImageJ-win64 software before and after the operation. The average SCV expansion rate was calculated to be 194.77 ± 30.38%. Pearson correlation analysis was performed to examine the relationship between the decrease in Oswestry Disability Index (ODI) score (indicating functional lumbar recovery) and the increase in DH and SCV expansion before and after the operation. The results showed a positive correlation between the ODI decrease value and both the DH increase (correlation coefficient *r *= 0.535, *p *= 0.033) and the SCV expansion rate (correlation coefficient *r *= 0.586, *p *= 0.017). This graph provides important insights into the correlation between functional lumbar recovery, DH increase, and SCV expansion in LSS patients after undergoing OLIF surgery. The positive correlations observed suggest that as the DH increases and the SCV expands, there is an improvement in functional lumbar recovery. These findings indirectly evaluate the safety and effectiveness of OLIF surgery for LSS patients. Overall, Fig. 3 highlights the positive relationship between functional outcomes, anatomical changes in disc height and spinal canal volume, and the efficacy of OLIF surgery in LSS patients (Fig. 3).

**Table 5:** Comparison of changes in preoperative and postoperative values of parameters of the disc height (DH), the cross-sectional area of vertebral canal (CSAVC), the cross-sectional area of dural sac (CSADS), the cross-sectional area of intervertebral foramen (CSAIF), and spinal canal volume (SCV)

Parameters	Preop (*n *= 16)	Postop (*n *= 16)	Value added	*t* value	*p* value
DH (mm)	9.58 ± 1.40	12.44 ± 1.38	2.85 ± 0.87	13.04	0.000
CSAVC (mm^2^)	95.39 ± 21.51	136.96 ± 19.08	41.57 ± 13.72	12.11	0.000
CSADS (mm^2^)	64.86 ± 16.26	94.01 ± 16.44	29.15 ± 12.23	9.53	0.002
CSAIF (mm^2^)	80.82 ± 15.39	107.75 ± 13.63	26.92 ± 9.08	11.84	0.000
SCV (mm^3^)	889.90 ± 177.50	1690.14 ± 205.22	800.24 ± 176.27	18.15	0.018

The measured values of DH, CSAVC, CSADS, CSAIF, and SCV after operation were significantly higher than those before operation (*p *< 0.05)

### One year postoperative return results for all parameters

After 1 year of follow-up postoperative, patients expressed satisfaction with the surgical efficacy. The clinical parameter results at 1 year postoperative were as follows (Table 5):

- Visual analogue scale (VAS) score: 1.91 ± 0.29
- Oswestry Disability Index (ODI): 23.28 ± 1.75%
- Lumbar lordosis (LL): 44.94 ± 6.11°
- Sagittal vertical axis (SVA): 27.24 ± 2.65 mm
- Disc height (DH) at 1 year postoperative: 11.87 ± 1.22 mm
- Cross-sectional area of the vertebral canal (CSAVC) at 1 year postoperative: 117.87 ± 14.69 mm.^2^
- Cross-sectional area of the disc (CSADC) at 1 year postoperative: 90.27 ± 16.12 mm.^2^
- Cross-sectional area of the intervertebral foramen (CSAIF) at 1 year postoperative: 103.75 ± 13.18 mm.^2^
- Spinal canal volume (SCV) at 1 year postoperative: 1389.11 ± 152.39 mm.^3^

The 1 year postoperative parameter values were compared to the preoperative values. Although there were some variations in the parameter values, there was still a significant difference compared to the preoperative values. These differences were statistically significant, indicating the long-term effectiveness of the surgery in improving clinical parameters and patient outcomes (Table 6).

**Table 6:** Comparison of parameter changes in visual analogue score (VAS), the Oswestry Disability Index (ODI), lumbar lordosis (LL), sagittal vertical axis (SVA), disc height (DH), the cross-sectional area of vertebral canal (CSAVC), the cross-sectional area of dural sac (CSADS), the cross-sectional area of intervertebral foramen (CSAIF), and spinal canal volume (SCV) parameters between preoperative and 1 year postoperative follow-up parameters

Parameters	Preop (*n *= 16)	1 year postop (*n *= 16)	*t* value	*p* value
VAS (score)	6.76 ± 0.59	1.91 ± 0.29	57.82	0.000
ODI (%)	67.93 ± 3.02	23.28 ± 1.75	95.77	0.000
LL (°)	34.6 ± 6.7	44.9 ± 6.1	13.09	0.000
SVA (mm)	53.6 ± 6.0	27.2 ± 2.7	− 23.57	0.001
DH (mm)	9.58 ± 1.40	11.87 ± 1.22	11.31	0.000
CSAVC (mm^2^)	95.39 ± 21.51	117.87 ± 14.69	6.96	0.000
CSADS (mm^2^)	64.86 ± 16.26	90.27 ± 16.12	8.28	0.002
CSAIF (mm^2^)	80.82 ± 15.39	103.75 ± 13.18	10.02	0.000
SCV (mm^3^)	889.90 ± 177.50	1389.11 ± 152.39	13.75	0.010

One year postoperative follow-up parameters VAS, ODI, LL, SVA, DH, CSAVC, CSADS, CSAIF, and SCV values were statistically significantly greater than preoperative parameter values (*P *< 0.05)

## Discussion

Lumbar spinal stenosis (LSS) is a common degenerative spinal condition. Currently, there is no widely accepted classification method for LSS. It is typically classified based on descriptive elements, aetiology (degenerative or congenital), location (primary, lateral recess, or intervertebral foramen), and severity (mild, moderate, and severe). However, further research is needed to establish a universally applicable classification system for LSS [12]. The OLIF procedure is commonly classified as applicable for mild to severe lumbar spinal stenosis (LSS). Mild to moderate spinal stenosis is typically characterized by a cross-sectional area of the dura mater between 75 and 100 mm, while severe spinal stenosis is defined as a cross-sectional area less than 75 mm. OLIF is not recommended for patients with congenital stenosis, bony stenosis, or space-occupying lesions in the spinal canal [11]. The development of mild, moderate, and severe spinal stenosis is often attributed to factors such as intervertebral disc herniation, thickening of the yellow ligament, and the proliferation and displacement of the upper facet joints in the lower vertebrae, resulting in foraminal stenosis [22]. These conditions lead to narrowing of the intervertebral foramina and spinal canal, reducing their volume, and causing compression of nerve roots and the dura mater, subsequently leading to neurological symptoms. OLIF is an indirect decompression method that involves creating a working channel through the natural space between the major psoas muscle and the abdominal great vascular sheath. This approach allows for the avoidance of critical nerves and blood vessels during the procedure [15]. In addition, by thoroughly removing the intervertebral disc in the surgical segment and performing bone grafting and fusion in that segment, and implanting an appropriately sized cage to fully restore the intervertebral space in the surgical area, the displaced facet joint is indirectly brought back to its normal anatomical position, thereby expanding the foraminal and spinal canal space. This process effectively enlarges the foraminal and spinal canal, reducing pressure on the nerves and alleviating symptoms.

In this study, a paired analysis was conducted to compare the preoperative and postoperative parameters of disc height (DH), cross-sectional area of the vertebral canal (CSAVC), cross-sectional area of the dural sac (CSADS), cross-sectional area of intervertebral foramen (CSAIF), and spinal canal volume (SCV). The results showed that all postoperative parameters were significantly improved compared to before the surgery, with a *p* value of less than 0.05, indicating statistical significance. Specifically, the average increase in disc height (DH) was 2.85 ± 0.87 mm, representing a 30% increase. The average increase in the cross-sectional area of the vertebral canal (CSAVC) was 41.57 ± 13.72 mm^2^, indicating a 43% increase. Additionally, the average increase in spinal canal volume (SCV) was 800.24 ± 176.27 mm^3^, representing a substantial 90% increase. These findings are consistent with the results reported by Cheng et al. [23], who also observed similar improvements in DH and CSA with the assistance of OLIF combined with pedicle screw internal fixation before and after the surgery. We conducted a thorough follow-up with all the patients 1 year after the operation, and we are pleased to report that the patients expressed satisfaction with the surgical efficacy. To evaluate the long-term effects, we analysed the follow-up parameters and compared them to the corresponding preoperative parameters as paired samples. Our findings revealed that the values of the parameters at 1 year after the operation demonstrated varying degrees of increase or decrease when compared to the immediate postoperative values. However, it is important to note that there remained a significant difference in the values of the parameters compared to the preoperative baseline, and this difference was statistically significant (*p *< 0.05). These results indicate that the improvements achieved through the OLIF procedure were sustained over the course of 1 year, providing continued benefits for the patients. Furthermore, we conducted an analysis of the line chart error for DH (disc height) and CSAVC (cross-sectional area of the vertebral canal) measurements in LSS (lumbar spinal stenosis) patients prior to the procedure, 1 month post-procedure, and 1 year post-procedure. The findings revealed a slight decrease of approximately 0.5 mm in DH 1 year after the operation compared to 1 month post-procedure, accompanied by a 19-mm reduction in CSAVC. These changes can be attributed to factors such as excessive fusion cage, endplate injury, and the additional rigidity provided by pedicle screw fixation. In this study, a total of three cases were observed, including two instances of fusion cage subsidence and one case of endplate injury. These findings are consistent with the results of Zeng et al. [24], who conducted a retrospective analysis of 235 cases of perioperative complications in OLIF patients. Their study reported 22 cases of endplate damage with a probability of 9.36% and 18 cases of cage subsidence and displacement with a probability of 7.66%, resulting in a combined probability of 17.02%. Similarly, Abe et al. [25] found that the most common perioperative complication in their retrospective study of 155 OLIF cases was endplate fracture/subsidence, with a probability of 18.7%. Therefore, based on the results of this study, we can compare the differences in DH (disc height), CSAVC (cross-sectional area of the vertebral canal), CSADS (cross-sectional area of the dural sac), CSAIF (cross-sectional area of the intervertebral foramen), and SCV (spinal canal volume) before and after the operation to assess the impact of OLIF on indirect decompression in LSS patients.

The results of this study indicate that the average intraoperative blood loss for patients is 43.1 ± 6.3 ml, and the average operative duration is 105.2 ± 7.4 min. These values are slightly higher compared to the intraoperative blood loss and operative time reported by Takayoshi Shimizu et al. [26]. However, it is important to note that this study focuses specifically on patients aged > 40 years old and examines the changes in the spinal canal before and after OLIF for narrow segments. The specific components of the operation are not specified in this study, whereas the previous study by Takayoshi Shimizu included a broader age range (> 20 years old) and a different surgical area. These factors may contribute to the slightly higher data results observed in this study. In this study, we conducted a comparison of VAS pain and ODI scores before and after the OLIF combined with pedicle screw surgery. The results showed that the measured values after the procedure were significantly lower than those before the operation (*P *< 0.05). This suggests that the symptoms of low back pain and intermittent claudication were considerably improved after the surgery. Furthermore, we performed a Pearson correlation analysis to examine the relationship between the decrease in ODI scores and the increase in DH and the expansion rate of SCV. The correlation coefficients for both comparisons were found to be statistically significant, with *r *= 0.535, *p *= 0.033 for the decrease in ODI and increase in DH, and *r *= 0.586, *p *= 0.017 for the decrease in ODI and expansion rate of SCV (*p *< 0.05). These findings indicate a correlation between the improvement in lumbar function and the surgical procedure. Additionally, the study demonstrated that the LL value increased significantly, and the SVA value decreased substantially after the operation (*P *< 0.01). These results suggest that OLIF combined with pedicle screw fixation offers advantages such as reduced trauma and shorter operation time in the treatment of patients with lumbar spinal stenosis. Moreover, it has a positive effect on the recovery of lumbar function and the restoration of sagittal balance in the spine.

The previous studies have primarily focused on single retrospective studies of oblique lumbar interbody fusion (OLIF) treatment for lumbar spinal stenosis (LSS), comparative studies between OLIF and other surgical methods, or diagnostic analysis through CT or MRI measurements of the dura mater [8, 27–30]. While existing studies have underscored the beneficial impact of OLIF surgery in treating patients with LSS, their primary focus has been on clinical parameters such as intraoperative blood loss, duration of surgery, and time taken to ambulate post-surgery. Even when radiological parameters were scrutinized, the analysis was largely limited to a cursory examination of sagittal sequence parameters such as disc height and lumbar lordotic angle. However, there remains a significant gap in the current research landscape concerning the changes in the spinal canal both pre- and post-surgery in patients with lumbar spinal stenosis (LSS) who have undergone oblique lumbar interbody fusion (OLIF) in combination with pedicle screw fixation. This is the crux of our study—to evaluate the indirect decompression effect of OLIF combined with pedicle screw fixation in treating LSS, as evidenced by changes in the spinal canal. In this study, we aimed to assess the indirect decompression effect of OLIF combined with pedicle screw internal fixation on LSS patients by comparing various parameters such as disc height (DH), cross-sectional area of the vertebral canal (CSAVC), cross-sectional area of the dural sac (CSADS), cross-sectional area of the intervertebral foramen (CSAIF), and spinal canal volume (SCV), as well as the SCV expansion rate. By analysing these parameters, we aimed to provide more data and explicit evidence regarding the indirect decompression achieved through OLIF combined with pedicle screw internal fixation. It is important to acknowledge that the small sample size in this study may limit the reliability of the results. Additionally, the absence of specific surgical segments and individual variations among patients could potentially affect result consistency. Furthermore, the follow-up observation was limited to a duration of only 1 year, while longer-term tracking could offer more compelling evidence. Therefore, further validation through larger sample sizes and comprehensive analysis of core data from multiple perspectives is still necessary.

## Conclusions

In summary, our study demonstrates that OLIF combined with pedicle screw fixation effectively restores intervertebral space height and increases the cross-sectional area of the spinal canal in LSS patients. These findings provide clear evidence of the indirect decompression effect achieved through OLIF combined with pedicle screw fixation. The parameters evaluated, including disc height (DH), cross-sectional area of the vertebral canal (CSAVC), cross-sectional area of the dural sac (CSADS), cross-sectional area of the intervertebral foramen (CSAIF), spinal canal volume (SCV), and SCV expansion rate, accurately reflect the changes in the spinal canal in LSS patients. These parameters hold significant value in assessing the efficacy of OLIF surgery in treating LSS and in evaluating the recovery of lumbar function in clinical practice.

## Data Availability

Raw data information for the results measured in this project is available from the first author or corresponding author upon reasonable request.
